# Hospital Charity: The Best Means of Preventing Its Abuse

**Published:** 1887-05-21

**Authors:** William Collier

**Affiliations:** Oxford


### Hospital Charity:

### THE BEST MEANS OF PREVENTING ITS ABUSE.

By William Collier, M.A., M.D., Oxford.

In the present paper I have made no attempt to bring forward proof of the existence of a widespread abuse of our great Medical Charities, because I believe that no one who is interested in or has a knowledge of the subject has ever denied its existence. I have endeavoured to describe the nature of such abuse; the evils to which it gives rise; and the best means of preventing it.

I. *The Nature of the Abuse.*—In dealing with this part of the subject, it is necessary to have a clear idea as to who is entitled to receive hospital aid. It will be generally admitted that "non-paupers who are unable to pay for adequate medical assistance when ill are entitled to it." I lay special stress on the word adequate, for under certain circumstances a patient might be abusing the charity, while under others he would be entitled to share its benefits. For example, a mechanic or small tradesman earning from 30*s*. to £2 a week, is attacked with a slight illness, which does not necessitate his giving up work; such a man is quite able either to become a member of a provident dispensary, or to pay a medical man small fees for attending him; if, however, his illness were prolonged for some weeks, or he met with a bad accident, preventing work, he would become a fit object for medical charity. Let me now point out a few classes of patients who make use of our hospitals, without having a fair claim on them.

1st. Well-to-do people, whose only reason for attending a hospital is to escape the payment of fees. Such cases are perfectly familiar to all physicians and surgeons who work among out-patients. It occasionally happens that people, who would not think of attending themselves, send their children under the care of their own servants to obtain gratuitous advice.

2nd. The servants of the rich. In many hospitals—I speak principally from a practical knowledge of provincial hospitals—it is a common practice for rich subscribers to give their own domestic servants, when ill, recommendations as out- or in-patients. The obvious objection to such a practice is, that the time of the physicians and surgeons is taken up and the hospital's funds used for a class of patients who are well able to pay the moderate fees of the local medical man, or should at any rate provide against illness by the payment of a small annual sum, which would enable them to ensure medical attendance when ill.

3rd. Accidents. It frequently happens that a person meets with an accident, and at once runs to the nearest hospital, where his wounds are attended to, and he is told to come again. He leaves the hospital without paying a fee, and quickly convinces himself that he is quite justified in attending until his wounds are healed; though probably, if taken ill, he would not think of seeking gratuitous medical advice. It would seem that in such a case the abuse does not so much depend on his seeking the hospital in the first place, but rather in his continued attendance, as by such attendance he may be making use of expensive surgical dressings, intended for the poor, and certainly depriving some medical man of fees to which he is entitled.

4th. The working man in receipt of good wages. In the present day great efforts are made to persuade the working man to subscribe to hospitals, the result too often being that he regards any small sum he may subscribe as giving him a perfect right to run to the hospital for assistance at the first approach of sickness. If the working man in the receipt of good wages is to be encouraged or allowed to seek hospital aid whenever ill, then undoubtedly a small fee should be paid for each visit, otherwise while at one moment we preach to the working man the virtues of thrift and self-help, at the next we encourage him to cast aside self-dependence and to rely entirely on charity.

5th. Another considerable class who seek, but are not entitled to hospital aid, are those who having once been treated, and perhaps quite rightly, at a hospital for a serious illness, in future always attend, however slight their ailment, their only reason being to escape expense.

II. *The Evils to which the Abuse gives rise.*—All who have worked among the poor have urged the necessity of self-help and thrift among them; the existing unchecked abuse of medical charities is doing much to choke the growth of such thrift. At the present time hospitals are struggling for existence, and in many cases overwhelmed with debt, consequently every farthing which goes towards the treatment of those able to pay is taken from the really poor. The well-to-do patient, by so drawing on the limited time at the disposal of the physician or surgeon, often renders it impossible for him to give to each case the time and attention it deserves, and may so be the means of inflicting a great injury on his poorer brothers. The overcrowded state of our out-patient rooms has long been a scandal, and is still so.

Lastly, the existing abuse inflicts very great hardships on the medical profession, who are deprived annually of large sums by the conversion into hospital patients of those who previously paid fees. Hospital physicians and surgeons suffer equally with their brethren, as patients learn to consult them at their hospitals instead of in their own consulting-rooms.

III. *The Best Means of Preventing the Abuse.*—Experience teaches that if we wish to put an end to any abuse, we must lose no opportunity of urging the necessity of the change on those who have it in their power to carry out the measures we advocate. The fact of our not meeting with much support or encouragement at first should make us more keen and unremitting in our efforts. I would urge, therefore, the absolute necessity of bringing before the charitable public the existence and nature of this widespread abuse. The mere fact of its being more widely recognised would, I doubt not, do much to lessen it. More particularly we should appeal to the leading members of the medical profession, for I fear we cannot hope to make much progress until they recognise more fully than they have in the past the great evils and gross injustice it fosters. Much good might be done if members of the medical profession could be induced to bring the subject forward for discussion at the various branch meetings of the British Medical Association We should especially endeavour to gain the ears of the committees of management of hospitals, as they could, and I believe would, if once induced to study the subject, lend us very valuable aid. In an earlier part of my paper I stated it as an axiom that only those who, without being absolute paupers, were unable when ill to pay for adequate medical advice, were entitled to hospital aid. This being so, the claim of each applicant for relief should be decided on its own merits. This would necessitate careful supervision and systematic inquiry in all hospitals. Within the past few months I have made inquiries as to the precautions adopted at most of our large provincial hospitals. In a few they seem admirable, in others hardly any exist.

The secretary of one hospital writes :—" The circumstances of every patient are inquired into by a clerk specially appointed to do so, and a second inquiry is also made in doubtful cases by an officer of the Provident Society, who visits patients residing in the town and sends me a report, which I then compare with the statements made to the clerk in the first instance. If any such patient is found to be receiving wages in excess of a fixed scale, he is refused further treatment at the hospital, and is referred to the Provident Dispensary of the district in which he resides." I need hardly add that if such precautions were very general, instead of being quite the exception, the necessity for remedial measures would scarcely exist.

The secretary of another large provincial hospital writes :—" It is against our rules for persons to make use of the institution who are able to pay for medical advice, but it is very difficult to discriminate, and we have to trust a good deal to the recommender."

I am afraid that trusting to the recommenders is the rule, with very few exceptions.

One of our largest London hospitals, the London, employs a paid officer to inquire into the circumstances of doubtful cases among the out-patients; while another, the Children's Hospital, Great Ormond Street, protects itself by refusing to give relief more than once in doubtful cases, until the applicant produces a letter from the Charity Organisation Society in the district in which he lives, stating that the case has been inquired into and is deserving of medical treatment.

If once the rule of investigating doubtful cases became general, the public would quickly learn that the medical aid of hospitals was only to be sought in cases of real necessity, and not as a means of saving their purses. To be successful, investigation must be carried out with tact and consideration. I would urge the necessity of allowing every applicant to see the physician or surgeon once, and of seeking their opinion before refusing further attendance, as a patient suffering from what was likely to be a long and tedious illness might well be a fit object for medical charity, while the same patient, if his illness were a slight one, might be quite the reverse. If such supervision was carried out with care and kindness, we should, I think, find very few genuine patients objecting to it. In many of the provincial hospitals such investigation might be undertaken by members of the committee of management in turn. In larger hospitals it would be necessary to appoint a special officer, whose duty it would be to report to the committee of management, or a sub-committee appointed for the purpose. In order to test the efficiency of such a plan, it is necessary that it should be generally adopted in London and other large towns where there are more hospitals than one.

It might seem at first sight that any such scheme would require a greater expenditure of time than members of committee would care to give. If such were the case, a paid officer should be employed; but it should be remembered that the duty of investigation would only necessitate a questioning of the patient as to his or her rate of wages, family, etc. Any further inquiry could be carried out by the hospital secretary, or under-secretary. Again, once enforce careful supervision, and in a very short time the number of doubtful applicants would quickly dwindle to very small dimensions.

I cannot conclude without discussing the question of the payment of small fees by out-patients. There will always be found in the out-patient rooms, patients who, having been under the treatment of their own doctors for a considerable time, are anxious for further advice. Many of these patients, while they are not able to pay the higher fees of the physicians and surgeons they wish to consult, can yet well afford to pay a small sum, from 1*s.* to 2*s.* 6*d.* for each visit. It has been proposed to enforce the payment of such fees from such patients.

While acknowledging that such a scheme would diminish the working expenses of our out-patient departments, and would do much to foster the spirit of self-dependence and thrift, yet, as the objections to it are very strong, and as the greater number of medical men would at present be opposed to so radical a change, I should not be prepared to support it very strongly, but think it is well worth a trial. One very strong objection to such a plan, is, that unless the strictest supervision be enforced, our general hospitals would be abused by a class of patients who do not at present use them, but who, under cover of a small fee, would soon learn to do so. Quite recently, at one of the London special hospitals, where small payments are made, I heard a patient complain of the expenses of his railway journey, stating that each visit to the hospital cost him 13*s.* 4*d.*, the town in which he lived being more than seventy miles from London. He appeared to me to be suffering from a disease that would probably have been equally well treated by a medical man in the town he left. The fact of getting a small operation performed, and his case treated by a London surgeon, with the payment of a very small fee for each attendance, probably induced this patient to seek the hospital. At another hospital I once heard a patient who was paying a shilling a fortnight, admit that he was in receipt of an income of £300.

Again, if the payment of small fees became generally enforced at hospitals, they might compete with the provident dispensaries, and so seriously weaken the efforts of those who in London and other large towns are endeavouring to establish thoroughly sound and well-managed provident institutions. I think one step in the right direction might be taken at once, by making many of the patients purchase their own cod-liver oil, and even their own drugs, direct from the chemist. The difficulty with regard to the purity and strength of the drugs supplied could be easily met by recommending a few chemists in the neighbourhood of the hospital, and having the drugs supplied occasionally tested.

In the present paper I have avoided dealing with the in-patient departments, because I believe the abuse of those departments is much less common and less important, and I considered it would be better for the purposes of discussion to confine ourselves to the greater evil.

To sum up. I would insist on the importance of bringing the subject as much before the eyes of the charitable public as possible. I would urge the necessity of endeavouring to induce hospital authorities, both lay and medical, to co-operate in an attempt to put an end to the abuses, isolated action being of very little use. And I would submit that at present more good would result from the adoption of a scheme involving a stricter and more careful supervision of patients, than by one which enforced the payment of small fees on the part of a certain number of the patients.

